# Indigenous populations health protection: A Canadian perspective

**DOI:** 10.1186/1471-2458-12-1098

**Published:** 2012-12-20

**Authors:** Katya L Richardson, Michelle S Driedger, Nick J Pizzi, Jianhong Wu, Seyed M Moghadas

**Affiliations:** 1 Centre for Disease Modelling, York Institute for Health Research, York University, Toronto, ON, M3J 1P3, Canada; 2 Department of Community Health Sciences, University of Manitoba, Winnipeg, MB, R3E 0W3, Canada; 3 Department of Computer Science, University of Manitoba, Winnipeg, MB, R3T 2N2, Canada

## Abstract

The disproportionate effects of the 2009 H1N1 pandemic on many Canadian Aboriginal communities have drawn attention to the vulnerability of these communities in terms of health outcomes in the face of emerging and reemerging infectious diseases. Exploring the particular challenges facing these communities is essential to improving public health planning. In alignment with the objectives of the Pandemic Influenza Outbreak Research Modelling (Pan-InfORM) team, a Canadian public health workshop was held at the Centre for Disease Modelling (CDM) to: (i) evaluate post-pandemic research findings; (ii) identify existing gaps in knowledge that have yet to be addressed through ongoing research and collaborative activities; and (iii) build upon existing partnerships within the research community to forge new collaborative links with Aboriginal health organizations. The workshop achieved its objectives in identifying main research findings and emerging information post pandemic, and highlighting key challenges that pose significant impediments to the health protection and promotion of Canadian Aboriginal populations. The health challenges faced by Canadian indigenous populations are unique and complex, and can only be addressed through active engagement with affected communities. The academic research community will need to develop a new interdisciplinary framework, building upon concepts from ‘Communities of Practice’, to ensure that the research priorities are identified and targeted, and the outcomes are translated into the context of community health to improve policy and practice.

## Introduction

The mandate of Pan-InfORM is to develop knowledge translation methodologies with the aim of bridging the gaps between theory, policy, and practice [1]. In a post-pandemic workshop held in 2010 [2]. Pan-InfORM analyzed public health and clinical responses to the 2009 H1N1 pandemic and found that Canada’s Aboriginal (First Nations, Inuit, and Métis) populations were disproportionately affected by the crisis. In fact, those living in First Nations communities were 2.8 times more likely to be hospitalized after contracting the infection, with an intensive care unit admission rate 3 times higher than non-Aboriginal people [3]. With a commitment to inform public health policies for the promotion of population health, Pan-InfORM has prioritized initiatives to address the challenges of community health in protecting vulnerable populations from emerging infectious diseases. In order to identify the pertinent challenges, a public health workshop on “Indigenous Populations Health Protection” was held on May 7-8, 2012 [4], at the CDM in York University. With the participation of key stakeholders from Aboriginal health organizations, policy decision-makers, and representatives from the research community in Canada, the workshop focused on public health responses, determinants of health, and the differential effects of intervention strategies in Aboriginal populations.

The presence of indigenous stakeholders was crucial in meeting the workshop objectives and providing a national forum to establish new partnerships, and foster research collaborations with Aboriginal health organizations. Modellers presented important research findings with relevance to indigenous health, and highlighted the importance of community-specific planning for vulnerable populations. From the standpoint of public health, the workshop uncovered some critical issues facing underserved communities in terms of access to healthcare services and program delivery. Representatives from health departments shared their knowledge and experiences with addressing the disparities in healthcare access for many Aboriginal communities across Canada. Through in-depth and collegial discussions, important inputs that must be encapsulated in modelling frameworks were identified, and the challenges that are involved in developing health policies were presented.

### Public health responses to emerging diseases

Aboriginal populations face different challenges during emerging infectious diseases. Some of these challenges result from limited access to and the delivery of health services, particularly when some of Canada’s constitutionally identified Aboriginal peoples have different levels of government responsible for the provision of healthcare. The federal government generally provides for First Nations and Inuit populations, whereas Métis citizens generally fall under provincial health jurisdictions. What compounds this, however, is that First Nations and Métis citizens might live quite geographically close, but experience differential access to healthcare and non-insured health coverage. These differences can begin to be eliminated through collaborative multi-jurisdictional efforts designed to address the health needs of affected individuals, particularly those living in remote areas.

**Figure 1:**
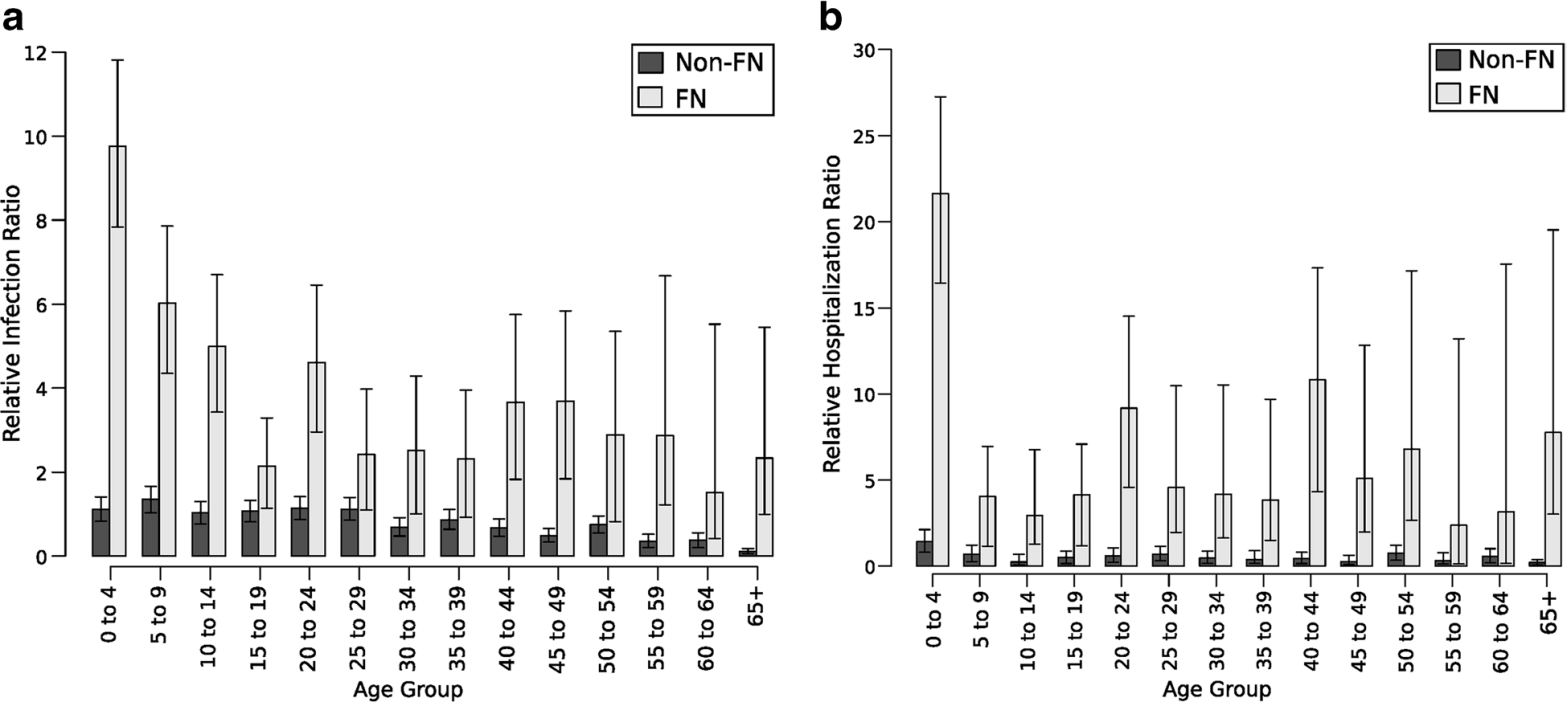
**(a) Relative infection ratios; and (b) relative hospitalization ratios for First Nations (FN) and non-First Nations (non-FN) age groups: Manitoba, Canada; Spring wave of the 2009 H1N1 pandemic.** For Relative infection (hospitalization) ratios, bar plots and 95% confidence intervals correspond to the age-standardized ratio of the proportion of infectious (hospitalized) cases in a given age group to the proportion of the population in the same age group [7]. A relative ratio higher than 1 indicates that the corresponding age group experienced a higher incidence of infection or hospitalization than the population as a whole.

Aboriginal communities in the province of Manitoba, especially in the northern region, were severely affected by the 2009 pandemic [5,6]. Data for laboratory confirmed cases of H1N1 infection and hospitalization collected during the first wave of the 2009 pandemic in the province of Manitoba suggest significantly higher age-specific rates of incidence and hospital admission for First Nations populations compared to non-First Nations populations (Figure 1).

During outbreaks of the H1N1 virus in Manitoba, a Tripartite table was established, which included representatives from the provincial and federal governments as well as representatives from First Nations and Métis self-governing organizations. The provincial minister of health liaised and dialogued regularly with the Tripartite table to develop communication strategies for access to primary healthcare in northern Manitoba. In addition, a table for ‘Equity and Ethics’ was established to collect feedback from communities and ensure that their respective citizens would receive equitable access to vaccine and other health resources. To facilitate responses, planning measures included the establishment of teams that were deployed to remote areas for the delivery of patient-care. Patients with severe outcomes requiring hospitalization were transported to the southern part of the province for access to critical care. However, indigenous leaders stressed the importance of developing self-care systems. In response to this concern, Manitoba Health aided with the distribution of H1N1 flu kits to communities where pharmacies and nursing stations were absent or not readily accessible. The provincial government also supported the use of traditional medicines and communicated the relevant information as to where such resources could be located.

Lessons learned from Manitoba’s experience during the H1N1 pandemic included understanding that organizations and communities have developed their own plans for responding to emerging crises, and this underscores the necessity for effective communications at all levels of the healthcare system and community for the development of a coherent strategy [8]. This was further highlighted by presenting the challenges that were encountered with the implementation of vaccination policies during the second wave, in particular with determining priority groups, eligibility criteria, and workforce requirements. Pandemic prevention strategies included recommendations to adopt methods for the impact assessment of major decisions on health inequalities, to increase the engagement between services and communities, to strengthen the vital role of families and communities, to promote a more equitable distribution of the determinants of health, and to enhance prevention programs and encourage more outreach. Several recommendations for enhancing pandemic preparedness at the provincial level were made, such as establishing recommended structures and elements for strategy development with an oversight body and a multi-stakeholder network. It was identified that there needs to be greater clarity in communicating policy guidelines, so that information is presented in a consistent and accurate manner to the public.

To enhance the perspectives of northern communities, several impediments to adequate healthcare delivery during the H1N1 pandemic in the territory of Nunavut were discussed. Cross-cultural barriers remain a key challenge in Nunavut, as many of the healthcare professionals practicing in the territory are often considered outsiders. There is a general lack of orientation to the territory for outside staff, especially with regards to language training. The learning of Nunavut’s two languages is not mandatory and classes are limited to basic training. Concerns regarding the potential to recruit and retain public health staff in Nunavut remain unaddressed. Nunavut still stores public health records in paper-based forms as opposed to electronic systems implemented in Canada at large, which causes problems in maintaining accurate records and surveillance systems. Although the need to generate and update these systems is known, vocalizing this concern has been limited. Furthermore, regulations that have been put in place to protect public health are often rendered obsolete as few people are trained in policing these regulations. The lack of adherence to, or inadequate level of compliance with some regulations has resulted in an increased health burden in the Inuit communities.

### Determinants of health

The social determinants of health can be categorized as: distal (historic, political, social and economic contexts), intermediate (community infrastructure, resources, systems and capacities), and proximal (health behaviours, physical and social environment). Colonization is a particularly important historical consideration, as are neo-colonial policies because they perpetuate discrimination and social exclusion, even into the twenty-first century. These processes hinder the development of healthy identities and self-esteem, and are ultimately responsible for poor mental and physical health. Chronic mental diseases affecting indigenous communities include schizophrenia, bipolar disorder and major depressive disorder, as well as post-traumatic stress disorder and addiction. Most astounding is the suicide rate among First Nations youth (aged 15–24), which is eight times the national average for females and five times the national average for males in Canada [9,10].

Other important social determinants of health include poverty, poor education, and overcrowded housing, which have a strong correlation to chronic and infectious diseases. The United Nations’ Human Development Index has been applied to Canada to understand the differences in quality of life and wellbeing between indigenous and non-indigenous populations [11]. Overall, Canada has consistently ranked within the top five nations in the world but when the socioeconomic status of Canadian Aboriginal peoples is factored in, Canada’s ranking on the index drops significantly. Many Aboriginal peoples are essentially living in Third World conditions within a First World country.

Geographic locale is a key factor in determining the level of access to healthcare, with the most underserved communities being those that are the most isolated and remote in the country. Often these communities belong to indigenous groups living in the northern regions of the provinces and across the territories. Approximately 78% of the Inuit population lives in Nunavut where resources pertaining to health practitioners and medical equipment are limited [12]. Geographic isolation complicates policy decisions, as the availability and lifespan of medicines need to be continually assessed to make accurate decisions about the shipment of medical supplies to remote communities. This was marked as one of the greatest challenges in serving these communities during the H1N1 pandemic outbreaks. Furthermore, the existence of multiple jurisdictions, each with differing policies concerning the health and wellbeing of First Nations has led to a patchwork of polices. Overlapping jurisdictions create conflicts in terms of identifying correct procedures to follow and assigning the responsibility for provision of care. Currently, the provincial and federal governments share these responsibilities and there is little consistency in the modes for public health delivery between regions, especially between on-reserve and off-reserve indigenous populations. The outcome has been varying levels of healthcare delivery, number of personnel, and facilities available within each indigenous community.

Recognizing that limited access to healthcare acts as one of the perpetuating factors to increased rates of respiratory illness in Aboriginal populations, a team of researchers has been conducting research to provide contrasting results if this limitation were removed [13,14]. The leading member of this research presented the findings specific to Kahnawà:ke, an Aboriginal reserve without any constraints in access to healthcare due to its close proximity to Montreal, the largest urban centre in the province of Quebec. Data was collected on outpatient and emergency room visits between 1996 to 2006 for residents of both Kahnawà:ke and Montreal. Analysis of such data, stratified by gender and age groups, indicates that the two regions had similar demographics, but the outpatient and emergency room visits were 33% higher for residents of Kahnawà:ke compared to residents of Montreal. When the access to healthcare factor was removed, questions arose about the reasons for the increased risk of vulnerability in residents of Kahnawà:ke. Although no conclusions have been drawn, data indicate that diabetes in First Nations populations is 70% higher than the Canadian national average; child obesity is 40% versus 23% in Quebec; and smoking is 50% higher in Quebec’s First Nations people compared to non-First Nations. A constant annual pattern has been reported, linking the contributing factors, habitual smoking, and obesity to respiratory illnesses.

Although the link between social factors and health outcomes is strong, Canada’s approach to protecting vulnerable populations has focused on emergency services rather than prevention. A study conducted at York University’s Canadian Homelessness Research Network analyzed how emergency response to homelessness impacts the vulnerability of homeless populations in the event of a pandemic, and how it presents impediments to effective pandemic planning [15]. This study challenged key assumptions made about the resources available to homeless populations and raised important questions about system capacities in the homelessness sector. It also analyzed ethical considerations, noting that during emergency situations, there is a risk of compromising human rights for greater health and safety. It is common for difficult ethical questions about the prioritization and allocation of limited health resources to arise during these occasions, as well as concerns about the violation of individuals’ autonomy through forced isolation or quarantine.

### Differential effects of intervention measures

The first wave of the 2009 H1N1 pandemic exposed the vulnerability of Aboriginal populations to poor outcomes, demonstrating the inefficacy of the polysaccharide vaccine that was in use at the time. This alerted Health Canada to fund a study that assessed the safety and immunogenicity of a new adjuvanted vaccine (Arepanrix) in a sample of Aboriginal adults [16]. A leading member of the study team presented the results of this population-specific work. The study involved an open trial with 138 participants, 95 with First Nations identity and 43 Métis, all from the Winnipeg health region, which is the largest urban centre in the province of Manitoba. The volunteers kept their daily symptom diaries for seven days following vaccination, including oral temperature measurements. There was a telephone-based safety interview on the seventh day, and an in-person review of adverse events on the twenty-first day. Approximately 70% of the volunteers experienced adverse effects, although fever was not experienced and most general symptoms were abated by the end of the first week. The immune response assessment involved collecting blood samples at baseline, as well as 21 to 28 days post vaccination. Sera were then tested for hemagglutination-inhibiting antibodies at the National Microbiology Laboratory in Winnipeg. All of the patients had adequate antibody responses regardless of whether they were primed (previously exposed) or naïve (fully susceptible). Results for the entire study, which examined 1,200 individuals across six projects, concluded that immunogenic responses to the vaccination in Aboriginal adults exceeded those of non-Aboriginal adults. Workshop participants found the protocol completion rate impressive, with 136 of the 138 subjects present for the final blood draw, which was greater than the anticipated rate of participation by Aboriginal people in the research. The safety profile of the adjuvanted vaccine was consistent with the projected rates. Given the success of this study, it will be important in future work to determine if an equally satisfactory response follows the adjuvanted seasonal influenza vaccine.

In the vaccine research domain, a study is currently being conducted for the development of a new vaccine candidate against *Heamophilus Infuenzae* type A (Hia), which has emerged during the past decade in Canadian Aboriginal communities [17-22]. The high prevalence of this infection, which manifests as meningitis, septicaemia, or bacteremic pneumonia, has prompted Aboriginal-specific studies. Invasive Hia disease has become a major cause of severe outcomes in young children of several Aboriginal populations in North America, with highest risk of contracting Hia being reported in the Navajo, White Apache, Alaskan natives, First Nations, and Inuit [17-20]. In a study spanning the last decade, it was found that Hia made up 45% of all serotyped isolates for *Heamophilus Infuenzae*[19]. The emergence and high incidence of invasive Hia disease in Canadian Aboriginal populations warrant further clinical and epidemiological studies, involving affected communities for the development of an effective Hia vaccine candidate.

Helicobacter pylori (H. pylori), is yet another prevalent infection in Canadian Aboriginal populations [23]. H. pylori is one of the most common pathogens affecting half of the world’s population, particularly in developing countries. In Canada, there are three identified groups, including Aboriginal people, which are associated with higher risk of infection by H. pylori [24]. The pathogen causes illnesses and conditions such as chronic gastritis and peptic ulcers, and increases the risk of gastric cancer [23-25]. The current infection management strategies are based on antibiotic regimens. However, this treatment faces declining effectiveness, with rates dropping below 80% due to the emergence of drug-resistance. A team of Canadian researchers has developed a new technology, which enables the formulation of a vaccine candidate against H. pylori. The leading member of the research team presented the results of this ongoing work that aims to analyze the characteristics of isolates from Aboriginal populations. This analysis could identify the circulating strains in Aboriginal populations, and determine the immune profiles of the affected populations. Factors responsible for the increased vulnerability of these populations to H. pylori include crowded housing, poor sanitary conditions, and polluted water supplies, which underscore the importance of access to critical infrastructure in protecting and promoting community health.

### Surveillance and modelling activities

A major obstacle to developing appropriate health policies and responsive healthcare delivery is a lack of specific data. Although data may be available for particular regions, there is a general lack of streamlining in data sets across multiple jurisdictions, as well as between hospital databases and public health surveillance systems in Canada. As a result, public health professionals are often faced with a deficiency of information to make informed policy decisions. Thus, incomplete data may be adapted or used out of the context in efforts to inform the development of programs and strategies.

During the workshop, disease modellers discussed some key areas in which detailed population level data play a critical role in understanding the risk of infection and outcomes in different vulnerable groups. For example, the results of a study on comparative analysis of age distribution of infection and hospitalization during the H1N1 outbreak, presented a marked difference in the risk of infection between First Nations and non-First Nations populations in Manitoba [26]. The study further discriminated between the first wave and second wave of the pandemic, and compared the incidence rates between on-reserve and off-reserve First Nation communities, indicating that the risk for infection and hospitalization was significantly higher for the former. Pre-school aged children in the First Nation populations were at higher risk during the first wave, whereas school-age children were at higher risk of infection during the second wave. Such comparative analysis was based on large databases created during the H1N1 pandemic with stratification of health regions, age, gender, ethnicity, time for initial care, and the type and duration of health resources used for the management of infection.

The need for detailed data is also important for the assessment of effectiveness and cost-effectiveness of intervention measures in the face of competing strategies. Presented results of an ongoing research study highlighted the potential utility of an agent-based modelling approach to determine the most effective antiviral treatment and prophylaxis strategies for influenza infection control, and to evaluate the effect of limiting intervention duration [27]. In summary, the preliminary findings suggested that a great deal of prophylaxis waste typically occurs at low treatment levels in early stages of the epidemic. The early administration of high treatment levels significantly reduces prophylaxis waste, but increasing prophylaxis coverage in some scenarios contributes to increased waste. Limiting the duration of prophylaxis can reduce the waste for comparable outcomes. This work continues to investigate whether particular age groups contribute disproportionately to the waste of treatment resources, and during which stage of the epidemic the greatest amount of waste is created. The results of this study will be used to propose more specifically targeted interventions that can be tested *insilico* using computer simulation experiments.

### Involvement with aboriginal communities

Workshop participants stressed the importance of developing relationships with Aboriginal stakeholders to ensure that their voices are heard in policy-making, and their needs are addressed in strategy development and program delivery. Yet, it can be difficult for researchers to honour all aspects of diversity in their work and use holistic and inclusive approaches, which equally weigh different systems of knowledge. To navigate these pertinent challenges, the use of a “Communities of Practice” (CoP) model was presented. The CoP model, defined as a group of people who have common concerns, a set of problems, and a passion about solving the problems [28,29], consists of two core components: (i) the interrelationships formed around mutual trust, identity, and understanding; and (ii) the acknowledgement of differences in perception and understanding. There is a need to develop shared meanings through social engagements and interactions by working towards a common goal and communicating in an accessible jargon-free language [30]. The concept of CoP is best summarized by the term coined by Mi’kmaw Elder Albert Marshall, “two-eyed seeing,” which refers to the ability to see with one eye from an indigenous perspective and with the other from a Western perspective, learning to use both in tandem for the benefit of all. This creates a comprehensive approach to advancing Aboriginal health objectives.

Within the context of CoP, the Director of the Institute of Aboriginal Peoples’ Health (IAPH) of the Canadian Institutes of Health Research (CIHR) outlined the institute’s two primary goals. First, the IAHP aims to increase awareness, understanding, and appreciation of Aboriginal beliefs, in addition to traditional knowledge among researchers, peer reviewers, and the Canadian population by extension. Second, the IAPH places a high priority on recognizing, promoting, and incorporating the excellence and rigour of methodologies derived from indigenous norms. The objective is to have these methodologies incorporated into the way the IAPH conducts its research, rather than remaining a mere side note. The IAPH aims to increase the number of First Nations, Inuit, and Métis researchers conducting Aboriginal-related health research, as well as the number and quality of their research activities. The IAPH also takes a grassroots approach in its support of community-based organizations that are eligible to receive and manage funds on behalf of the CIHR, thereby increasing the communities’ abilities to address their own health issues.

### Discussion session

The workshop highlighted three general areas of research that are neither discrete nor inclusive but can be labeled as instrumental, symbolic, or conceptual. Instrumental research measures impact, symbolic research argues a position, and conceptual research evaluates whether the right questions are being formulated. Each form of research has its own relevancy and can be more commonly associated with certain academic disciplines. For example, with respect to the topic of health risks among homeless populations, instrumental research may ask how shelters can be made more hygienic, while conceptual research would question whether it makes more sense to provide housing to homeless individuals rather than warehouse them in shelters. Both types of research are important for public health and through interdisciplinary knowledge translation activities, the pertinent questions can be debated, re-framed and re-formulated so that they are meaningful and address public health concerns. The underlying process is complex and there will always be political challenges involved; however it is important that resources be marshaled to address these public health issues to produce maximum benefits to the communities at risk.

With unique population characteristics that place some Aboriginal communities at increased risk for adverse health consequences, it is imperative for public health authorities to identify vulnerable segments of the population, and in cooperation with local officials within the community, determine effective and feasible health responses. These responses must also take into account factors such as jurisdictional issues and the variability of Aboriginal public health infrastructures. While effectiveness is a necessary criterion for the identification of optimal health responses, these factors must also be considered for assessing the feasibility of such responses in different community settings. Taking into consideration these underappreciated aspects and realities of vulnerable populations, the workshop highlighted that modelling and simulations are invaluable tools that permit the rapid testing of hypotheses for the subsequent design and implementation of response strategies to address the needs of these populations. By permitting simultaneous observations of disease-related outcomes at multiple levels of communities and the healthcare system, models can inform the development of community-wide and specific contingency plans that incorporate the full spectrum of harms related to disease spread and benefits associated with response activities.

## Conclusions

The workshop was successful in bringing together key stakeholders, policy decision makers, and researchers from a wide array of disciplines, each with their own perspective but all with the common goal of improving the health status of Canada’s indigenous populations. During the preceding decade, Canada has experienced the emergence of novel diseases that have caused tremendous public concern and economic consequences, including the 2003 SARS epidemic and the 2009 influenza pandemic. The rapid containment of SARS as the first major infectious disease threat of the 21^st^ century was a public health success in the modern era [31], but also a warning that the global containment of emerging diseases may be much more difficult in this highly connected world, if not impossible. The 2009 influenza pandemic demonstrated this difficulty and incurred disproportionately large economic and political impacts, in addition to differential effects on many subpopulations including Aboriginal peoples and underserved communities [3]. While the focus here has been on First Nations, Inuit and Métis people in Canada, these experiences were similar to what happened for Indigenous people living in the United States, Australia and New Zealand [32-34].

The objectives of this networking event, and the spectrum of participants, attested to the fact that in the relatively short period of time since the inception of Pan-Inform, significant progress has been achieved through the hosting of bi-annual workshops of this scale. These networking activities have encouraged more intricate disciplinary dialogues, which challenge participants to re-evaluate their assumptions so that they are eventually resolved or dissolved. As a result of such interdisciplinary approaches, new and stronger links between theory, policy and practice have been forged. A strength of the workshop was the presentation of how appropriate use of data can lead to novel scientific findings that influence policy and practice. This is realized by involving the research community, affected populations, and policy makers in the interpretation and contextual use of data, which is becoming increasingly important as modellers aim to introduce ever more complex structures into the models such as social network patterns. Public health challenges and research methods discussed during the workshop led to key recommendations outlined in Table 1.

**Table 1:** Summary of workshop discussion and recommendations

**Topics**	**Type of disease**	**Research methods**	**Recommendations**
Vaccines	Helicobacter pylori, Haemophilus influenzae	Laboratory, surveillance, and modelling	Develop new vaccine candidates for helicobacter pylori and haemophilus influenza A
Antiviral drugs	Influenza viruses with pandemic potential	Modelling and simulations	Evaluate antiviral strategies for emerging influenza viruses in remote and isolated communities. Create formal structures for strategy development with oversight bodies and multi-stakeholder networks
Healthcare access, program development, and delivery	Infectious diseases (influenza, haemophilus influenzae, helicobacter pylori, tuberculosis, sexually transmitted diseases)	Population health surveillance, modelling and simulations	Enhance collaborative multi-jurisdictional efforts. Involve indigenous communities in the development of healthcare programs and delivery. Deploy more resources (e.g., medical equipment and pharmaceutical measures) to remote regions and improve training of healthcare professionals. Streamline public health surveillance systems across Canada to build more comprehensive databases
Determinants of health	Chronic diseases (cancer, cardiovascular diseases, diabetes); Chronic mental health illnesses (schizophrenia, bipolar disorder, major depressive disorder, post-traumatic stress disorders)	Population health surveys, clinical investigations, community engagement, and focus group discussions	Develop educational programs to eliminate cross-cultural barriers (e.g., language training). Review and evaluate the impact of public health decisions on health inequalities. Develop policies and programs to address homelessness beyond responses to emerging crises

Presentations given during the workshop were evidence for the opening of a new chapter in Canadian public health research and practice involving indigenous populations. Ongoing studies for the development of vaccines for diseases to which Aboriginal populations are prone, as well as projects that are population specific and function on community engagement are examples of movements towards addressing indigenous populations health protection. In moving forward, research should be integrated with planning, building capacity, and harmonizing response activities at all levels and across the healthcare system to help develop holistic policies that are context-specific and incorporate indigenous perspectives.

## Competing interests

The authors declare that they have no competing interests.

## Authors’ contributions

KR and SM wrote the first Draft of this paper. MD, NP and JW contributed to the final version. All authors have read the paper and approved it.

## Ethics

Data represented in Figure 1 was approved by the Human Research Ethics Board of the University of Manitoba, and Health Information Privacy Committee of Manitoba.

## Pre-publication history

The pre-publication history for this paper can be accessed here:

http://www.biomedcentral.com/1471-2458/12/1098/prepub
